# Role of sleep quality in the acceleration of biological aging and its potential for preventive interaction on air pollution insults: Findings from the UK Biobank cohort

**DOI:** 10.1111/acel.13610

**Published:** 2022-04-14

**Authors:** Xu Gao, Ninghao Huang, Xinbiao Guo, Tao Huang

**Affiliations:** ^1^ 12465 Department of Occupational and Environmental Health Sciences School of Public Health Peking University Beijing China; ^2^ 12465 Department of Epidemiology and Biostatistics School of Public Health Peking University Beijing China

**Keywords:** aging, air pollution, biological age, PM_2.5_, sleep

## Abstract

Sleep has been associated with aging and relevant health outcomes, but the causal relationship remains inconclusive. In this study, we investigated the associations of sleep behaviors with biological ages (BAs) among 363,886 middle and elderly adults from UK Biobank. Sleep index (0 [worst]–6 [best]) of each participant was retrieved from the following six sleep behaviors: snoring, chronotype, daytime sleepiness, sleep duration, insomnia, and difficulties in getting up. Two BAs, the KDM‐biological age and PhenoAge, were estimated by corresponding algorithms based on clinical traits, and their residual discrepancies with chronological age were defined as the age accelerations (AAs). We first observed negative associations between the sleep index and the two AAs, and demonstrated that the change of AAs could be the consequence of sleep quality using Mendelian randomization with genetic risk scores of sleep index and BAs. Particularly, a one‐unit increase in sleep index was associated with 0.104‐ and 0.119‐year decreases in KDM‐biological AA and PhenoAge acceleration, respectively. Air pollution is another key driver of aging. We further observed significant independent and joint effects of sleep and air pollution (PM_2.5_ and NO_2_) on AAs. Sleep quality also showed a modifying effect on the associations of elevated PM_2.5_ and NO_2_ levels with accelerated AAs. For instance, an interquartile range increase in PM_2.5_ level was associated with 0.009‐, 0.044‐, and 0.074‐year increase in PhenoAge acceleration among people with high (5–6), medium (3–4), and low (0–2) sleep index, respectively. Our findings elucidate that better sleep quality could lessen accelerated biological aging resulting from air pollution.

## Graphical abstract

Our study explored the causal relationship between sleep and aging, which remains inconclusive. Sleep quality was negatively associated with the accelerations of two biological ages (BAs) in 363,886 UK adults. Mendelian randomization demonstrated that worsening sleep quality could accelerate BAs. Sleep and air pollution (i.e., PM_2.5_ and NO_2_) could accelerate two BAs independently. Better sleep quality could lessen the accelerations of two BAs resulting from exogenous exposures such as PM_2.5_ and NO_2_.

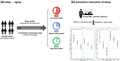

## 1 INTRODUCTION

Aging is a gradual and progressive deterioration in biological system integrity, which is thought to arise from the accumulation changes at the cellular level (Ferrucci et al., 2018). It is accompanied by changes in sleep quality, quantity, and architecture, especially in elderly adults (Carroll & Prather, 2021). Nevertheless, the mutual causal association between sleep and accelerated aging is still in debate. Along with many physiological alterations in normal aging, sleep behaviors change with aging independent of many factors including medical comorbidity and medications (Li et al., 2018). Some researchers thus suggest that sleep disorders are the consequence of aging‐related changes in neuroendocrine function (Li et al., 2018). Meanwhile, sleep is also considered a restorative process that not only allows for energy renewal but also for cellular restoration (Carroll & Prather, 2021). Hence, the other hypothesis is that the declines in sleep quality may lead to accelerated aging by triggering DNA damage and chronic inflammation to influence the compensatory/resiliency systems of the human body (Carroll & Prather, 2021). The lack of an established accurate measurement of aging, however, hinders the scientists from elucidating the directionality and causal relationship between aging and sleep.

Aging is the sum of changes that occur at hierarchically organized levels in the human body (Ferrucci et al., 2018), which makes it hard to be captured by single age‐related biomarkers employed in previous relevant studies. All individuals age chronologically at the same rate, but there is marked variation in their biological ages as we observe in real life that the people with the same chronological age may not share the same aging‐related symptoms (Ferrucci et al., 2018). Some could experience age‐related decline faster than others. Moreover, as biological aging is a complex biological process in multiple organ systems, a single aging‐related biomarker, such as telomere length or oxidative stress biomarkers, may not be able to completely depict the whole landscape of the aging process of individuals due to the heterogeneity of cells (Lopez‐Otin et al., 2013). Therefore, the identification of “biological age (BA)” has been proposed and has been explored over the last 10 years. To date, several forms of BAs which could be estimated based on the functions of cardiovascular, metabolic, renal, immune, and pulmonary systems (e.g., KDM‐biological age (Klemera & Doubal, 2006; Levine, 2013) and PhenoAge (Levine et al., 2018)), or based on aging‐related DNA methylation profiles (known as “epigenetic clocks” (Horvath & Raj, 2018)), have been developed. Their discrepancies with chronological age, that is, the age accelerations (AAs), have been highly associated with aging‐related health outcomes and mortality (Horvath & Raj, 2018). However, previous studies on the associations of sleep with BAs or aging‐related symptoms usually employed only one or two sleep behaviors or were conducted in a specific population with limited participants (Carroll et al., 2017, 2021; Carskadon et al., 2019; Han et al., 2018; Sun et al., 2020). Therefore, there is a dearth of studies exploring the associations between sleep and aging in larger populations with more detailed sleep information, and causal inference approaches are needed to uncover their causal nature explicitly.

Additionally, air pollution, especially the fine particulate matter [PM < 2.5 μm (PM_2.5_)], is a critical environmental exposure that could advance aging (Peters et al., 2021) and affect sleep quality (Liu et al., 2020). Previous studies have linked aberrant accelerated epigenetic clocks with the increased exposure to various air pollutants in different populations (Peters et al., 2021). Plenty of evidence has also documented increased risks of sleep disorders (Liu et al., 2020) and sleep‐related neurological impairments, for example, dementia and cognitive decline (Gao et al., 2021; Schikowski & Altug, 2020), associated with elevated air pollution levels. Nevertheless, since the direction of the sleep‐aging relationship is still undetermined, no studies yet evaluated whether the sleep quality or BAs could modify or mediate the adverse effects of air pollution on biological aging or sleep quality, which is critical for developing interventions to mitigate the adverse effects of air pollution.

Therefore, we examined the causal associations of sleep (as reflected by six sleep behaviors) with KDM‐biological age and PhenoAge based on the measures of clinical traits of multiple organs in the UK Biobank, a national‐wide population‐based cohort study in the UK. We subsequently explored the associations of five major air pollutants (PM_2.5,_ PM with an aerodynamic diameter between 2.5 and 10 µm [PM_coarse_], PM with an aerodynamic diameter of less than 10 µm [PM_10_], nitrogen dioxide [NO_2_], and nitrogen oxides [NOx]) with AAs and sleep, and explored whether AAs could mediate or modify the associations of air pollution with sleep, or whether the AAs could reflect the predisposition of participants regarding the impact of air pollution on their sleep quality.

## 2 RESULTS

### 2.1 Participants’ characteristics and air pollution distributions

Table 1 presents the baseline characteristics of 363,886 study participants by sleep index category. Participants’ age (mean ± standard deviation) was 56.5 ± 8.1 years and most of them were white. About 35% and 55% participants were former and never smokers, respectively. The majority of them had healthy physical activity and >10 years of education. Nearly half were with a healthy daily intake of alcohol. Only about 24%, 5%, and 5.5% participants were with hypertension, diabetes, and CHD diagnosed by doctors, respectively. Insomnia complaint is the most frequent (~75%) sleep‐related behavior that the participants had and most participants (~82%) could get up easily in the mornings. About 30% have a high sleep quality (sleep index = 5–6) and 15% have a low sleep quality (sleep index = 0–2). The high sleep quality group has lower BAs and higher AAs than the low sleep group, and the medium sleep quality group (sleep index = 3–4) has the BAs and AAs at the intermediate level in between. Both BAs were highly correlated with the other and with the chronological age (Figure S1). The average concentrations of air pollutants were 9.96 ± 1.05 μg/m^3^ (interquartile range [IQR] = 1.27) for PM_2.5_, 6.42 ± 0.90 μg/m^3^ (IQR = 0.79) for PM_coarse_, 19.23 ± 2.01 μg/m^3^ (IQR = 2.33) for PM_10_, 28.93 ± 9.10 μg/m^3^ (IQR = 10.80) for NO_2_, and 43.58 ± 15.47 μg/m^3^ (IQR = 16.44) for NOx. Levels of all air pollutants were highly correlated (Table S1, all *p*‐values <0.001).

**TABLE 1:** Baseline characteristics of all participants and by sleep index category^a^

Characteristic	All (*n* = 363,886)	Sleep index category		
		Low (index = 0–2) (*n* = 55,174)	Medium (index = 3–4) (*n* = 201,795)	High (index = 5–6) (*n* = 106,917)
Age (years)	56.52 (8.07)	56.13 (8.02)	56.88 (7.97)	56.04 (8.23)
BMI (kg/m^2^)	27.39 (4.74)	28.78 (5.39)	27.48 (4.68)	26.51 (4.30)
Sex (male)	163,532 (44.9%)	25,765 (46.7%)	92,222 (45.7%)	45,545 (42.6%)
Race (white)	345,349 (94.9%)	50,998 (92.4%)	191,918 (95.1%)	102,433 (95.8%)
Smoking status^b^				
Current smoker	37,016 (10.2%)	8361 (15.2%)	20,715 (10.3%)	7940 (7.5%)
Former smoker	128,074 (35.3%)	20,204 (36.7%)	73,092 (36.3%)	34,778 (32.6%)
Never smoker	197,734 (54.5%)	26,432 (48.1%)	107,378 (53.4%)	63,924 (59.9%)
Healthy alcohol intake (yes)^c^	181,313 (49.9%)	25,002 (45.4%)	99,870 (49.5%)	56,441 (52.8%)
Healthy physical activity (yes)^d^	256,498 (71.7%)	34,491 (64.1%)	141,816 (71.5%)	80,191 (75.9%)
Years of education (>10 years)	236,963 (65.1%)	33,833 (61.3%)	129,472 (64.2%)	73,658 (68.9%)
Major diseases^e^				
Hypertension	86,758 (23.8%)	15,475 (28.1%)	49,993 (24.8%)	21,290 (19.9%)
Diabetes	17,883 (4.9%)	4270 (7.7%)	9972 (4.9%)	3641 (3.4%)
Coronary heart disease	19,931 (5.5%)	4439 (8.1%)	11,253 (5.6%)	4239 (4.0%)
Biological ages				
KDM‐biological age	49.11 (11.87)	49.57 (11.68%)	49.43 (11.84)	48.26 (11.96)
KDM‐biological age acceleration	−7.41 (9.09)	−6.56 (8.96)	−7.45 (9.10)	−7.78 (9.11)
PhenoAge	47.70 (9.73)	46.19 (9.77)	45.92 (9.62)	44.42 (9.79)
PhenoAge acceleration	−8.82 (5.34)	−9.94 (5.50)	−10.96 (5.09)	−11.62 (4.89)
Components of biological ages				
FEV_1_ (L)^f^	2.80 (0.74)	2.75 (0.74)	2.80 (0.74)	2.84 (0.74)
SBP (mm Hg)^f^	139.64 (19.35)	139.10 (19.06)	140.12 (19.34)	139.04 (19.50)
Total cholesterol (mg/dl)^f^	220.46 (42.92)	218.88 (44.34)	220.85 (43.14)	220.55 (41.74)
Glycated hemoglobin (%)^f^	5.44 (0.56)	5.52 (0.66)	5.44 (0.56)	5.38 (0.50)
Blood urea nitrogen (mg/dl)^f^	15.10 (3.64)	15.06 (3.83)	15.14 (3.65)	15.03 (3.53)
Lymphocyte (%)^g^	28.98 (7.28)	28.91 (7.40)	28.98 (7.29)	29.02 (7.21)
Mean cell volume (fl)^g^	82.78 (5.17)	82.73 (5.31)	82.79 (5.17)	82.78 (5.11)
Serum glucose (mg/dl)^g^	91.40 (17.95)	92.82 (21.14)	91.46 (17.91)	90.55 (16.09)
Red cell distribution width (%)^g^	13.46 (0.89)	13.53 (0.95)	13.46 (0.88)	13.43 (0.88)
White blood cell count (1000 cells/μl)^g^	6.86 (1.76)	7.05 (1.86)	6.87 (1.77)	6.74 (1.69)
Albumin (g/dl)^f^, ^g^	4.52 (0.24)	4.51 (0.25)	4.52 (0.24)	4.53 (0.24)
Creatinine (mg/dl)^f^, ^g^	0.81 (0.17)	0.82 (0.17)	0.81 (0.17)	0.81 (0.16)
C‐reactive protein (mg/dl)^f^, ^g^	0.25 (0.41)	0.30 (0.47)	0.25 (0.41)	0.22 (0.37)
Alkaline phosphatase (U/L)^f^, ^g^	83.04 (23.9)	85.50 (25.36)	83.34 (23.66)	81.22 (23.42)
Sleep behaviors for sleep index				
No self‐reported snoring (behavior 1)	227,927 (62.6%)	17,018 (30.8%)	116,296 (57.6%)	94,613 (88.5%)
Early chronotype (behavior 2)	229,411 (63.0%)	11,256 (20.4%)	120,434 (59.7%)	97,721 (91.4%)
No frequent daytime sleepiness (behavior 3)	277,377 (76.2%)	22,947 (41.6%)	151,197 (74.9%)	103,233 (96.6%)
Sleep 7–8 h/day (behavior 4)	248,038 (68.2%)	17,552 (31.8%)	128,967 (63.9%)	101,519 (95.0%)
Never/rarely insomnia (behavior 5)	88,533 (24.3%)	2925 (5.3%)	32,888 (16.3%)	52,720 (49.3%)
Getting up easy in morning (behavior 6)	299,436 (82.3%)	21,529 (39.0%)	171,919 (85.2%)	105,988 (99.1%)
Sleep index (continuous)	3.77 (1.23)	1.69 (0.54)	3.58 (0.49)	5.19 (0.40)

^a^ Mean values (standard deviation) for continuous variables and *n* (%) for categorical variables.

^b^ Data missing in 1062 participants.

^c^ Healthy alcohol intake: male: <28g/day; female: <14g/day; data missing in 23 participants.

^d^ Healthy physical activity: ≥150 min/week moderate or ≥75 min/week vigorous or 150 min/week mixed (moderate + vigorous) activity; data missing in 6066 participants.

^e^ Major disease diagnosed by doctor.

^f^ Employed to construct KDM‐biological age

^g^ Employed to construct PhenoAge.

### 2.2 Associations of sleep with biological ages

**FIGURE 1:**
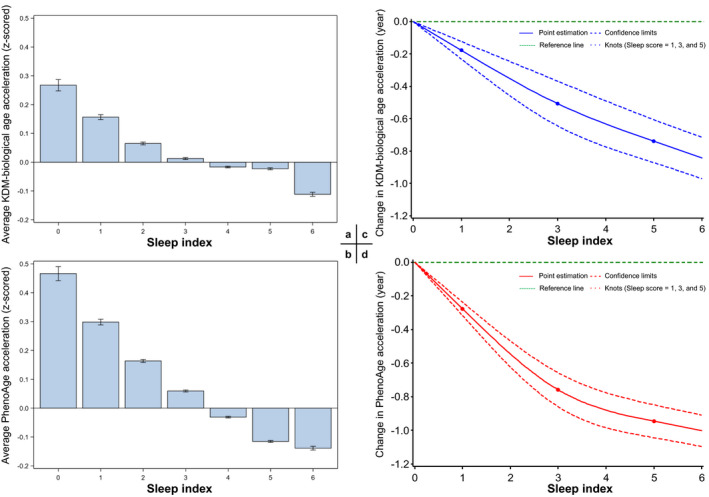
Averaged age accelerations by sleep index and best‐fitting dose‐response curves. In panels a and b, the blue bars are the mean values of *z*‐scored age accelerations by the sleep index, error bars are the standard deviations of the age accelerations; in panels c and d, the solid lines are the point estimations, the blue/red dash lines are the 95% confidence limits, the green dash lines are the reference lines, and the dots are the knots at sleep score = 1, 3, and 5

We first examined the associations of both forms of AAs with each of the six sleep behaviors (Table 2). After controlling for all potential covariates, we found that four and five sleep behaviors were negatively associated with KDM‐biological AA and PhenoAge acceleration, respectively. Early chronotype and normal sleep duration were significantly associated with both AAs. Early chronotype was associated with 0.071‐ and 0.234‐year decreases in KDM‐biological AA and PhenoAge acceleration, respectively. And, normal sleep duration was associated with 0.245‐ and 0.206‐year decreases in KDM‐biological AA and PhenoAge acceleration, respectively. KDM‐biological AA was additionally associated with self‐reported snoring and insomnia, and PhenoAge acceleration was additionally related to frequent daytime sleepiness and difficulties in getting up in the mornings. We further observed significant negative associations between the continuous sleep index and both AAs (Table 2 and Figure 1a,b). One unit increase in the sleep index was associated with 0.104‐ and 0.119‐year decreases in KDM‐biological AA and PhenoAge acceleration, respectively. The AAs of the high sleep quality group were 0.335 (KDM)‐ and 0.443‐year (PhenoAge) lower than the low sleep quality group, and the changes in both AAs of the medium sleep quality group were at the intermediate levels. As illustrated in Figure 1c,d, monotonic negative dose‐response relationships between continuous sleep index and both AAs were observed. Sensitivity analysis in psychiatric illness‐free participants showed slightly attenuated but still robust associations (Table S2), which suggests that the influence of underlying psychiatric illness on the primary findings might be minor.

**TABLE 2:** Associations of six sleep behaviors and sleep index with the accelerations of biological ages^a^

Sleep behaviors		KDM‐biological age acceleration (years)		PhenoAge acceleration (years)	
		Coefficients (*SE*)	*p*‐Value	Coefficients (*SE*)	*p*‐Value
Self‐reported snoring	No	−0.287 (0.023)	**<0.0001**	0.065 (0.067)	0.29
	Yes	Ref		Ref	
Chronotype	Early	−0.071 (0.022)	**0.0015**	−0.234 (0.017)	**<0.0001**
	Later	Ref		Ref	
Frequent daytime sleepiness	No	−0.015 (0.025)	0.56	−0.191 (0.019)	**<0.0001**
	Yes	Ref		Ref	
Sleep duration	Normal (7–8 h)	−0.245 (0.023)	**<0.0001**	−0.206 (0.017)	**<0.0001**
	Short or long	Ref		Ref	
Insomnia	Never or rarely	−0.079 (0.025)	**0.0018**	−0.029 (0.019)	0.12
	Sometimes or often	Ref		Ref	
Getting up in morning	Very or fairly easy	−0.009 (0.029)	0.76	−0.341 (0.021)	**<0.0001**
	Not or not very easy	Ref		Ref	
Sleep index (continuous, 0–6)		−0.104 (0.009)	**<0.0001**	−0.119 (0.007)	**<0.0001**
Sleep index (category)	High (5–6)	−0.335 (0.034)	**<0.0001**	−0.443 (0.025)	**<0.0001**
	Medium (3–4)	−0.179 (0.031)	**<0.0001**	−0.319 (0.023)	**<0.0001**
	Low (0–2)	Ref		Ref	

^a^ Model adjusted for age, sex, race, BMI, smoking status, healthy alcohol intake, healthy physical activity, years of education (<10 years or ≥10 years), hypertension, diabetes, and coronary heart disease. The examination center was controlled for as a random effect to account for the potential residual bias from examinations. Bolded values that were below the significance threshold, which was 0.05/(8*2) = 0.0031, were considered as statistically significant.

Furthermore, to investigate the causal associations between sleep and AAs, we conducted two MR analyses in two directions. Constructed genetic risk scores (GRSs) have the acceptable variance explained ranging from ~2.3% (for sleep duration) to ~51% (for KDM‐biological AA) for the MR analyses (Table S3). As demonstrated in Table 3 with unweighted GRSs for sleep index and AAs, we observed significant negative associations between genetic‐predicted sleep index and both AAs. However, the genetic‐predicted KDM‐biological AA was positively associated with sleep index, and the negative association between genetic‐predicted PhenoAge acceleration and sleep index was not statistically significant. Similar trends were also observed for each of the six sleep behaviors (Table S4) and another sensitivity analysis with weighted GRSs of sleep additionally demonstrated similar trends for each scenario (Table S5). Altogether, these results indicate that sleep quality was more likely to be the determinant of the change in AAs rather than a consequence.

**TABLE 3:** Causal associations between sleep index and biological age accelerations with unweighted genetic risk scores^a^

	KDM‐biological age acceleration (years)		PhenoAge acceleration (years)	
	Coefficients (SE)	*p*‐Value	Coefficients (SE)	*p*‐Value
(1) Sleep index → Biological ages				
Observed effects	−0.129 (0.011)	**<0.0001**	−0.153 (0.008)	**<0.0001**
Genetic‐predicted effects	−1.654 (0.037)	**<0.0001**	−0.808 (0.028)	**<0.0001**
(2) Biological ages → sleep index				
Observed effects	−0.034 (0.003)	**<0.0001**	−0.044 (0.002)	**<0.0001**
Genetic‐predicted effects	0.099 (0.068)	0.15	0.008 (0.011)	0.48

^a^ Effects were estimated by one SD change in the sleep index or biological age accelerations. Models were adjusted for age, sex, race, BMI, smoking status, healthy alcohol intake, healthy physical activity, years of education (<10 years or ≥10 years), hypertension, diabetes, and coronary heart disease. The examination center was controlled for as a random effect. The genetic risk scores for sleep index and biological age accelerations were unweighted. Bolded values that were below the significance threshold, which was 0.05/(2*2*2) = 0.00625, were considered as statistically significant.

### 2.3 Joint effects of air pollution and sleep on biological ages

**FIGURE 2:**
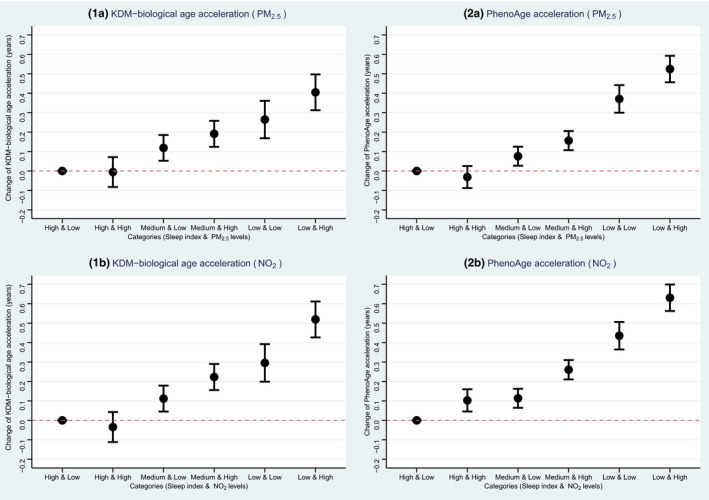
Joint associations of sleep index and air pollution levels with biological age accelerations. The dots are point estimations and the error bars are the 95% confidence limits

Given it was more plausible that accelerated AAs were the consequences of impaired sleep quality, along with the known association between air pollution and biological aging, we validated the associations of the five air pollutants with each AA and explored the hypothesis that whether sleep index could mediate the effects of air pollutants on AAs. As shown in Table S6, PM_2.5_ and NO_2_ were the two air pollutants that were significantly associated with both AAs in models without the adjustment of sleep index. An IQR increase in PM_2.5_ level was associated with 0.060‐ and 0.042‐year higher KDM‐biological AA and PhenoAge acceleration (*p*‐values < 0.0001), respectively. The same change in NO_2_ level was associated with 0.097‐ and 0.093‐year higher KDM‐biological AA and PhenoAge acceleration (*p*‐values < 0.0001), respectively. Their effects and *p*‐values were slightly reduced in models which additionally controlled for sleep index. Meanwhile, the effects of sleep index on both AAs were also marginally altered in this mutual adjustment model. To understand their joint effects explicitly, we classified the participants based on binary air pollution levels (by median) and sleep index category, and then observed clear and stepwise increasing trends of the joint effects of both factors on the two AAs (Figure 2). Particularly, compared to the group with high sleep quality and lower exposure to PM_2.5_, people with low sleep quality and higher exposure had a 0.397‐ and 0.496‐year higher KDM‐biological AA and PhenoAge acceleration (*p*‐values < 0.0001), respectively (Table S7).

### 2.4 Modifying effects of sleep quality

**FIGURE 3:**
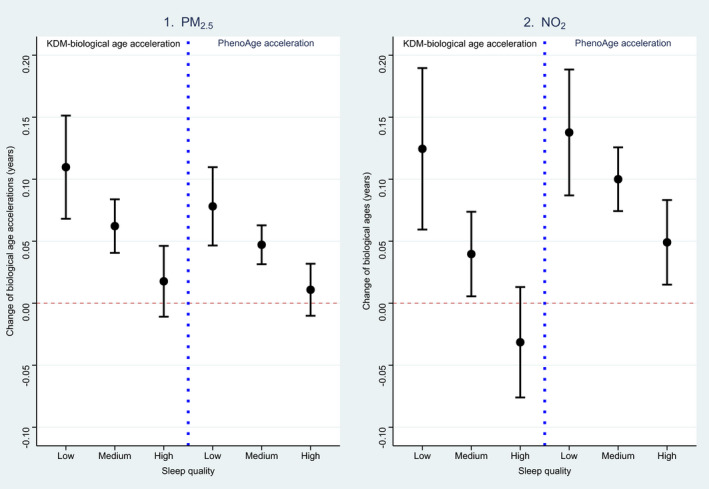
Associations of air pollution levels with biological age accelerations by sleep quality. The dots are point estimations and the error bars are the 95% confidence limits

**FIGURE 4:**
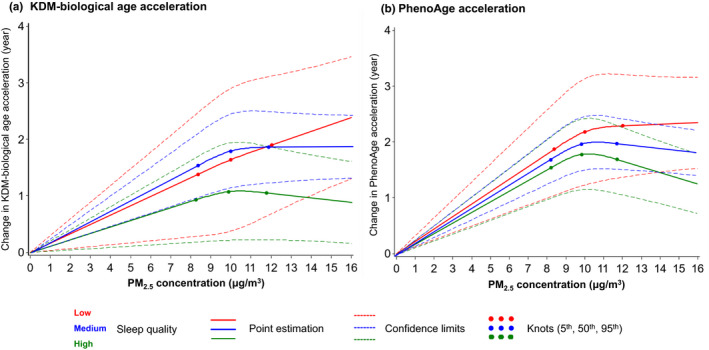
Best‐fitting models for the relationships of PM_2.5_ exposure with accelerations of two biological ages, by sleep quality. The solid lines are the point estimations, the dash lines are the 95% confidence limits, and the dots are the knots at 5th, 50th, and 95th percentiles of PM_2.5_ concentration. Red, blue, and green colors represent low, medium, and high sleep quality, respectively

**FIGURE 5:**
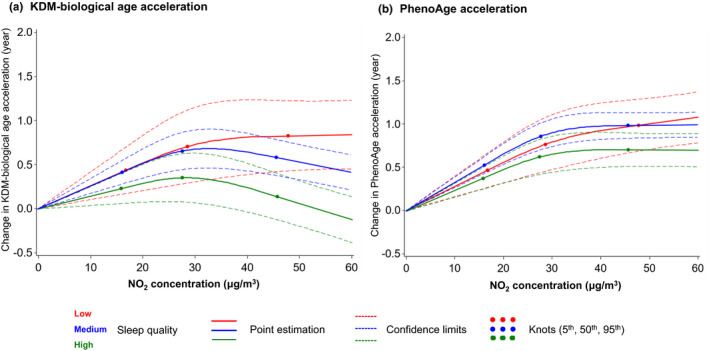
Best‐fitting models for the relationships of NO_2_ exposure with accelerations of two biological ages, by sleep quality. The solid lines are the point estimations, the dash lines are the 95% confidence limits, and the dots are the knots at 5th, 50th, and 95th percentiles of NO_2_ concentration. Red, blue, and green colors represent low, medium, and high sleep quality, respectively

According to the robust joint effects of air pollution and sleep on AAs, we additionally explored the potential modifying effect of sleep quality on the associations between air pollution and AAs since sleep is a modifiable factor that could be intervened by human behaviors relatively easily. As shown in Figure 3 and Table S8, we observed a robust modifying effect of sleep quality on the air pollution‐AA associations (*p*‐values of interaction terms <0.001). Particularly, an IQR increase in PM_2.5_ concentration was associated with 0.009‐, 0.044‐, and 0.074‐year increase in PhenoAge acceleration among people with high, medium, and low sleep quality, respectively. The same increase in NO_2_ concentration was associated with 0.048‐, 0.098‐, and 0.133‐year increase in PhenoAge acceleration among people with different sleep qualities. Even though not all interactions of each sleep pattern with air pollutants were statistically significant (Table S9), a clear trend was observed that participants with healthier sleep patterns were with less accelerated AAs under the exposure of both pollutants compared to those without healthier patterns. We further evaluated the linearities and dose‐response relationships of the PM_2.5_ and NO_2_ with both AAs. Non‐linear relationships for each air pollutant were observed (Figures 4 and 5). Modifying effects of sleep quality on the changes in both biological AAs could be distinguished under relatively lower PM_2.5_ (~10 μg/m^3^, Figure 4) and NO_2_ levels (~30 μg/m^3^, Figure 5).

## 3 DISCUSSION

In this large cohort of middle‐ and elderly‐aged adults, we demonstrated that worsening sleep quality could accelerate biological aging with BAs estimated by two well‐established algorithms and a comprehensive sleep index combining the impacts of six major sleep disorders. We further observed that people with low sleep quality and higher exposure to PM_2.5_ or NO_2_ had the highest AAs. Sleep quality could also modify the associations of elevated PM_2.5_ and NO_2_ levels with accelerated aging. For instance, an IQR increase in PM_2.5_ concentration was associated with 0.009‐, 0.044‐, and 0.074‐year increase in PhenoAge acceleration among people with high, medium, and low sleep index, respectively.

To date, our study is the first investigation demonstrating that impaired sleep quality could accelerate human aging in a large population with the state‐of‐art of causal interpretation. Our observed negative sleep‐BA association has been partially suggested by previous studies. In Han et al.’s study, they reported a positive association of sleep duration of >8 h/day with an increased BA estimated based on five phenotypes (Han et al., 2018), which was also found in our study that abnormal sleep duration was associated with increased AAs. Additionally, Carroll et al. and Carskadon et al. separately linked insomnia and insufficient sleep duration with accelerated epigenetic clocks among older females (Carroll et al., 2017), pregnant women (Carroll et al., 2021), and freshmen (Carskadon et al., 2019), respectively. In line with these studies, we observed increasing patterns of AAs under worsening general sleep quality. Previous studies also provided much more marginal evidence that is consistent with our findings with respect to other aging biomarkers. For example, in a study of physicians, overnight on‐call participants had lower baseline DNA repair gene expression and more DNA breaks than participants who did not work overnight (Cheung et al., 2019). And, shortened telomere length, a well‐known biomarker of cellular aging, was also found in relation to <6 h of sleep (Liang et al., 2011) and chronic poor sleep quality (Cribbet et al., 2014), respectively. Sun et al. (2020) also preliminarily found that poor sleep was associated with being frail, another aging‐related syndrome. Taken together, these complex aging biomarkers including BAs provided an exciting new avenue for investigating the underlying biological mechanisms of the interplay between sleep‐ and aging‐related health outcomes. We additionally found that four behaviors showed inconsistent associations with the two AAs. Such unique patterns might be explained by that the behaviors may be more closely related to the unique features of each BA as the KDM‐biological age is inclined to reflect the loss of system integrity while PhenoAge is more related to the risk of mortality (Hastings et al., 2019; Parker et al., 2020).

More intriguingly, although aging is tied to sleep difficulties firmly, due to the relatively limited sample size and cross‐sectional nature, previous studies were not able to address the causal associations. Our study answered this scientific question of interest by a causal inference test using the MR scheme. We found that it was more likely that sleep behaviors lead to the changes in AAs with statistically significant associations between genetic‐predicted sleep index and both AAs. This suggests that sleep quality may affect the aging of organs and systems reflected by two BAs based on mainly blood biochemistry biomarkers, and indicates that improving modifiable aspects of sleep may help to lessen the adverse effects associated with biological aging and to attenuate the risks of aging‐related diseases. Since BAs have been associated with cardiovascular health (Zhong et al., 2016), our finding is in agreement with that sleep quality is a preventable risk factor of cardiovascular events, which have been well‐established and recognized in large population‐based studies and clinical trials (Covassin & Singh, 2016). However, our study did not fully exclude the possibility that aging could be one of the major determinants of sleep impairment. Given the GRSs of sleep patterns could only explain 2.25%–8.1% variances in sleep patterns (Table S3), multiple factors including environmental, social, lifestyle, medical, and psychiatric conditions may contribute to elevated AA and their contributions could not be captured by the genetic background (Lopez‐Otin et al., 2013). Explained variances for the GRSs of AAs were much higher than that of sleep patterns since selected SNPs were retrieved from the same cohort.

Furthermore, literatures have established the adverse impact of air pollution on sleep quality in different perspectives (Liu et al., 2020), such as the risks of breathing problems, insomnia, sleep efficiency, and overall sleep quality, as well as on aging in different populations (Peters et al., 2021). With the previously determined causal association between sleep and AAs in the first step, we expected to find a robust mediation effect of sleep on the air pollution‐AA relationship. However, we observed very limited changes in the effects of different air pollutants on AAs in models mutually adjusting for sleep index, which suggests that sleep does not play a major role in mediating the effects of air pollution on the two AAs. Since sleep is predominantly related to cognitive health and brain aging (Li et al., 2018), especially the structural and physiological changes that occur in the brain, such limited mediation effects could be explained by the minimal capacity of the two BAs in measuring aging‐related neurological changes. The clinical traits implemented in the constructions of the two BAs were not specifically associated with such abnormal alterations related to the central neuron system. This is in line with the findings of our sensitivity analysis in psychiatric illness‐free participants, suggesting that the two BAs may not be closely related to the aging‐related damage of the neurological systems. Instead, we observed prominent modifying effects of sleep on the air pollution‐BA relationship, which implies that a healthy and adequate sleep may help attenuate the adverse aging effects of air pollution on non‐neurological systems (Figure S2). Also, we noted response curves with different features of the PM_2.5_ and NO_2_ with both AAs. Because KDM‐biological age is more related to the capacity and function of systems and organs (Hägg et al., 2019), and PhenoAge is skewed to predict the mortality risk of humans (Levine et al., 2018). Such various features of the modifying effects of sleep on the AAs suggest that sleep may help lessen the detrimental impacts of PM_2.5_ and NO_2_ on body functions at a lower exposure level and could also attenuate the lethal effects of the two pollutants on mortality when they reached a higher level.

Regarding the underlying biological mechanisms under the interesting modifying effects of sleep, one of the potential explanations is the reduction of oxidative stress and inflammation during healthy and adequate sleep. First, air pollutants can induce oxidative stress, the ability to respond to which has been identified as a key determinant of biological aging (Peters et al., 2021). Sleep may help with the anti‐oxidative mechanism by removing reactive oxygen species resulting from air pollution insults (Atrooz & Salim, 2020). But given our BAs were constructed based on nonoxidative biomarkers, this hypothesis could be validated in future biological studies. Beyond this, sleep may also help enhance immune defenses and stabilize the dysregulation of inflammatory responses under air pollution exposure. Previous studies have suggested that sleep impairment is associated with increased serum levels of C‐reactive protein (CRP), interleukin (IL)‐1, IL‐6, IL‐17, tumor necrosis factor α, and nuclear factor‐kappa B in addition to alteration of numbers and activity of macrophages and natural killer cells (Atrooz & Salim, 2020; Meier‐Ewert et al., 2004). The aberrant changes in such systematic inflammation biomarkers have been found to be involved in the impact of air pollution on the integrity of organs and the development of aging‐related diseases (Wu et al., 2018). This could be a more possible biological mechanism as our BAs have components related to inflammation such as lymphocyte proportion and CRP.

The major strengths of this study include the large sample size and relatively sufficient phenotype and biochemistry data for the biological age estimation. Several limitations are notable when interpreting the results. First, UK Biobank is a volunteer cohort, and participants are likely healthier than the general population, which may limit the effect of sleep on BAs in our analysis as their AAs are expected to be lower than the general population theoretically. Furthermore, the measurement bias of air pollution must be noted. The air pollution data we used were mostly a single measurement of the annual average outdoor air pollution level in 2010 since the home addresses of the participants were unavailable during follow‐up. Because the initial assessment visit of UK Biobank was from 2006 to 2010, we were unable to determine the lag or short‐term (<1 month) effects of air pollution on both sleep and AAs. Also, as most individuals spend a large amount of time indoors, individual exposure to all forms of air pollution may differ from that indicated by the ambient outdoor levels we used. Additionally, as sleep data used in our analyses were self‐reported, misclassification of sleep behaviors might exist, and the contributions of each included behavior cannot be weighted due to the lack of relevant studies. Such bias may attenuate our findings toward the null and underestimate the effects we observed. Meanwhile, our sleep index dichotomized six sleep behaviors for simplicity rather than including all sleep behaviors or measuring the changes of sleep behaviors before and after the survey, which may cause residual bias to some extent. Both aforementioned limitations highlight the need for future studies with comprehensive measures of sleep quality with well‐established questionnaires to confidently validate our findings of this study. Cautious is recommended to interpret our main results as an exploratory finding as the sleep index is yet validated clinically in comparison with other established sleep scales including Pittsburgh sleep quality index (PSQI) or Ford Insomnia response to stress Test (FIRST). Also, as a study with a cross‐sectional nature, even though the MR could make causal inference to somewhat extent, caution must still be taken in the causal interpretation on sleep and aging. And, given the genome‐wide association studies (GWAS) we used to build the GRS for BAs is the only available one but was conducted in UK Biobank, these SNPs we selected may be biased and not objective adequately. Therefore, we conducted a sensitivity analysis using 11 SNPs reported in a GWAS of DNA methylation age to create the GRS for both BAs/AAs (Gibson et al., 2019) as the DNA methylation age has been highly associated with the two BAs we investigated in this study (Belsky et al., 2020). The findings were in line with our primary findings by showing that sleep quality was more likely to be the determinant of the change in AAs rather than a consequence (Table S10). Last, participants in this study were mostly of European descent, which limits the generalization of our findings to other races.

In conclusion, our study is the first identifying the accelerating effect of poor sleep quality on biological aging. With this premise, we further found that sleep and air pollution were independently associated with biological aging, and sleep quality may modify the aging effects of air pollution. These findings not only provide exploratory evidence supporting sleep as an aging contributor but also underscore the importance of high‐quality sleep as an intervention approach to mitigate the negative impact of air pollution on human aging. Nevertheless, aging is associated with a myriad of changes in psychological, social, spiritual, financial, and lifestyle across the lifetime (Lopez‐Otin et al., 2013), and the sleep index we used also needs clinical validations. Further longitudinal studies with a more detailed evaluation of aging and comprehensive clinical measures of sleep quality are highly warranted to validate our findings and further determine the underlying biological mechanisms.

## 4 EXPERIMENTAL PROCEDURES

### 4.1 Study design and population

Study design and methods of UK Biobank have been reported in detail previously (Sudlow et al., 2015). In brief, UK Biobank is a large‐scale prospective study with 502,536 participants aged 37–73 years recruited in 2006–2010 with multiple follow‐ups. At the initial visit, participants provided information on sleep and other health‐related aspects through touch‐screen questionnaires and physical measurements. Blood samples were collected for genotyping and biochemistry tests. UK Biobank research has approval from the North West Multicenter Research Ethical Committee. All participants provided written informed consent for the study. In this analysis, we included 363,886 participants with available data of sleep behaviors, measures of biological traits for BA construction, and air pollution. This report followed the Strengthening the Reporting of Observational Studies in Epidemiology (STROBE) reporting guideline. This research has been conducted using the UK Biobank Resource under Application Number 44430.

### 4.2 Assessment of sleep behaviors

Due to the lack of specialized questionnaires of sleep, such as PSQI or FIRST at the baseline survey of UK Biobank, we instead used an algorithm based on self‐reported sleep quality information that was first introduced in 2020 for UK Biobank cohort (Fan et al., 2020). This algorithm has been used widely since 2020 and was used to make an index for sleep with 5 sleep‐related items (Fan et al., 2020; Li, Zheng, et al., 2021; Li, Xue, et al., 2021) or 4 items (Sambou et al., 2021), and showed the capacity as an alternative approach to reflect the sleep patterns of the participants of UK Biobank. Therefore, to optimize the sleep quality assessment in the UK Biobank, we used six self‐reported sleep behaviors in this study: snoring, chronotype, daytime sleepiness, sleep duration, and insomnia that were used in 5‐item sleep score (Fan et al., 2020) and the “difficulties in getting up in the morning” pattern that was found to be related to the risk of terminated health‐span (Sambou et al., 2021) to enhance the measurement of sleep quality and the capacity of the below‐mentioned sleep index (Table S11). Detailed assessment of sleep behaviors can be found in supplementary methods. According to the six sleep behaviors, we generated a sleep index. The low‐risk categories of each component were no self‐reported snoring, early chronotype (“morning” or “morning than evening”), no frequent daytime sleepiness (“never/rarely” or “sometimes”), normal sleep duration (7–8 h/day), reported never or rarely having insomnia symptoms, and getting up easy in morning (“fairly easy” or “very easy”). For each sleep behavior, the participant received a score of 1 if he or she was classified as the low‐risk group or 0 if otherwise as the high‐risk group. All component scores were summed to obtain a continuous sleep index ranging from 0 (worst) to 6 (best), with a higher index indicating a general better sleep quality. We further defined a sleep index category as high (5–6), medium (3–4), and low (0–2) based on the continuous sleep index.

### 4.3 Assessment of biological ages

We computed the BAs derived from a total of 12 blood chemistry traits, systolic blood pressure, and lung function data (Table S11) with two commonly accepted algorithms, the Klemera–Doubal method (i.e., KDM) and the PhenoAge method. Both algorithms were initially trained in data from the National Health and Nutrition Examination Survey (NHANES) following the method originally described by Klemera et al. (Klemera & Doubal, 2006; Levine, 2013) and Levine et al. (2018) with two sets of nine clinical traits (Table 1). The two BAs were constructed with different purposes. KDM‐biological age was computed from an algorithm derived from a series of regressions of nine individual biomarkers on chronological age in the reference population to quantify the decline of system integrity, and PhenoAge was computed from an algorithm derived from multivariate analysis of mortality hazards to estimate the risk of death (Hastings et al., 2019; Parker et al., 2020). The two BAs were developed in the white population (Hastings et al., 2019) and were stable in other populations and cohorts (Parker et al., 2020). The selected traits, algorithms, and corresponding R code can be found in the R package ‘BioAge’ at: https://github.com/dayoonkwon/BioAge and corresponding publications (Belsky et al., 2020; Kwon & Belsky, 2021). Missing values of each trait consisted of <10% of all traits and were, therefore, imputed by the median value of the corresponding trait. The residual differences between the estimated BAs and chronological age were considered as AAs since this approach could minimize the heterogeneities between the measurement platforms of each component of BAs (Hägg et al., 2019; Horvath & Raj, 2018). The residuals were calculated by a linear regression procedure in which one of the BAs was the outcome and chronological age was the independent variable. AAs were the targeted outcomes/modifiers in our primary analyses.

### 4.4 Exposure assessments

As previously described (Wang et al., 2021), the annual average concentrations of PM_2.5,_ PM_coarse_, PM_10_, NO_2_, and NOx were calculated centrally by the UK Biobank using a Land Use Regression (LUR) model developed by the ESCAPE project. More details on the air pollution data of the UK Biobank cohort and LUR model can be found at: http://biobank.ndph.ox.ac.uk/showcase/label.cgi?id=114 and in the supplement methods. The exposure data of PM_2.5_, PM_coarse_, and NOx were collected in 2010, while annual concentrations of NO_2_ and PM_10_ were available for several years (2005, 2006, 2007, and 2010 for NO_2,_ and 2007 and 2010 for PM_10_). Since the baseline survey of UK Biobank was conducted from 2006 to 2010, to achieve a better risk prediction, averaged values of NO_2_ and PM_10_ were included in the analysis.

### 4.5 Measurements of covariates

We included age, sex, body mass index (BMI), race, smoking status, healthy alcohol intake status, healthy physical activity status, years of education (<10 years), and prevalent hypertension, coronary heart disease (CHD), and diabetes that could be associated with aging as well as sleep quality as covariates to address potential confounding. Height and weight were measured by trained nurses during the baseline assessment center visit, and BMI was calculated by dividing weight in kilograms by the square of height in meters. Healthy alcohol intake status was defined as: male: <28 g/day; female: <14 g/day. Healthy physical activity status was defined as: ≥150 min/week moderate or ≥75 min/week vigorous or 150 min/week mixed (moderate + vigorous) activity. The Metabolic Equivalent Task (MET) minutes based on items from the short International Physical Activity Questionnaire (IPAQ) was adopted to assess physical activity. The history of hypertension and diabetes was based on self‐reported information and medical records.

### 4.6 Construction of genetic risk scores of sleep index and biological ages

Detailed information on genotyping, imputation, and quality control in the UK Biobank study has been described previously (Sudlow et al., 2015). We created GRSs for each sleep behavior, sleep index, and both AAs for the MR analyses. A total of 583 single‐nucleotide polymorphisms (SNPs) based on the up‐to‐date largest GWAS of the six sleep behaviors in ~1.3 million participants from 23andMe and UK Biobank cohorts were selected for the GRS of six sleep behaviors and sleep index (reported *p*‐value < 5 × 10^−8^) (Jansen et al., 2019). For the GRS of AAs, we used the latest and the only GWAS on both BAs (Kuo et al., 2021), which, respectively, reported 16 and 29 SNPs that were robustly associated with KDM‐biological age and PhenoAge (reported *p*‐value <5 × 10^−8^). Because the SNPs were evaluated in pruned genetic data in previous studies, no further loci pruning was conducted in our study. Each selected SNP was recoded as 0, 1, or 2 according to the number of risk alleles, and missing SNP values of individuals were imputed. The unweighted GRSs were directly summed up and the weighted GRSs were calculated by summing up after multiplied with its effect value, and then divided by half of the total effect size. The detailed SNPs were demonstrated in Table S12. The unweighted GRSs were employed in the primary analyses and the weighted ones for sleep index and AAs were used in the sensitivity analyses.

### 4.7 Statistical analysis

We first examined the associations of each sleep behavior and the sleep index with both forms of AAs using mixed‐effect linear regression models in which the AAs were the outcomes. We adjusted for covariates described in the previous section including chronic diseases and additionally controlled for the examination center in the model as a random effect to account for the potential residual bias from examinations. Since psychiatric disorders may affect sleep quality, we additionally conducted a sensitivity analysis in 285,054 participants that were free of dementia, depression, and anxiety. These disorders were ascertained using hospital inpatient records and Patient Health Questionnaire (PHQ)‐4 questionnaire as previously described (Milaneschi et al., 2021; Zhang et al., 2021).

Then, we performed one‐stage MR analyses to explore the causal relationships between sleep and BAs in two scenarios using mixed‐effect linear regression models. The GRS of sleep index was used to predict sleep index genetically and to study the effect of sleep on BAs. The GRS of BA was used to predict AAs genetically and to study the effects of the two AAs on sleep quality. The statistically significant genetic‐predicted effect in either scenario that was in the same direction as the observed effect in the primary model would indicate the plausibility of a causal effect. Corresponding dose‐response curves between sleep index and the two BAs were further assessed by restricted cubic spline regression (Desquilbet & Mariotti, 2010). Models were adjusted for the previously described covariates and the sleep index = 1, 3, and 5 were selected as knots.

Based on the results of MR analyses, the next part of our study would test whether (a) sleep quality could mediate or modify the association between elevated air pollution levels and accelerated AAs (i.e., if declined sleep index →the increased AAs) or (b) AAs could mediate or modify the association between elevated air pollution levels and declined sleep quality (i.e., if the increased AAs →declined sleep index). The possibility of the mediator role of the factors of interest would be examined by comparing the estimates of air pollutants in mixed‐effect linear regression models with air pollutants only with the ones in models with the mutual adjustment of both air pollutants and the potential mediator. If a robust mediation effect was identified, the mediation proportion would be estimated by the mediation analysis using the SAS function “PROC CAUSALMED”. Meanwhile, the possible modifying effects of the factors of interest would be examined by an interaction term of air pollution and the factor in a mixed‐effect linear regression model adjusting for corresponding air pollution and factors. If the interaction terms were statistically significant and hence suggested a likely modifying effect, a subgroup analysis by sleep index category or binary AAs (by median) would be conducted to test the effects of air pollution on AAs or sleep quality in a mixed‐effect linear regression model. The effects of air pollutants in all models were demonstrated by one IQR increase in the concentrations. Corresponding dose‐response curves and linearity of the relationships between air pollutants and BAs by sleep index category were subsequently assessed by restricted cubic spline regression if necessary. The 5th, 50th, and 95th percentiles were selected as knots. The ranges of air pollutants for curves were set according to the air quality guidelines of the WHO (World Health Organization, 2021) and their distributions in the UK Biobank cohort.

SAS version 9.4 TS1M5 (SAS Institute Inc.) was used to conduct data cleaning and all analyses. A two‐sided *p*‐value of <0.05 was considered statistically significant. Bonferroni corrections were conducted to correct the multiple comparisons according to the number of tests in each step of analysis accordingly.

## CONFLICT OF INTEREST

None reported.

## AUTHOR CONTRIBUTIONS

Xu Gao conceptualized the study, conducted data clean, estimated biological ages, and draft and reviewed the manuscript; Ninghao Huang coordinated the data collection, reviewed and revised the manuscript; Xinbiao Guo critically reviewed and revised the manuscript; Tao Huang coordinated and supervised the data collection, and reviewed and revised the manuscript.

## Supporting information

Fig S1

Fig S2

Table S1‐S11

Table S12

Supplementary Material

## Data Availability

Data are available in a public, open access repository. This research has been conducted using the UK Biobank Resource under Application Number 44430. The UK Biobank data are available on application to the UK Biobank (www.ukbiobank.ac.uk/).
