# Supplementary material for: Role of sleep quality in the acceleration of biological aging and its potential for preventive interaction on air pollution insults: Findings from the UK Biobank cohort
Source: Aging Cell. 2022 Apr 14;21(5):e13610. doi: 10.1111/acel.13610 (PMC9124313; doi:10.1111/acel.13610)
Supplement: Supplementary file 2 — Fig S2 [file ACEL-21-e13610-s001.pdf]

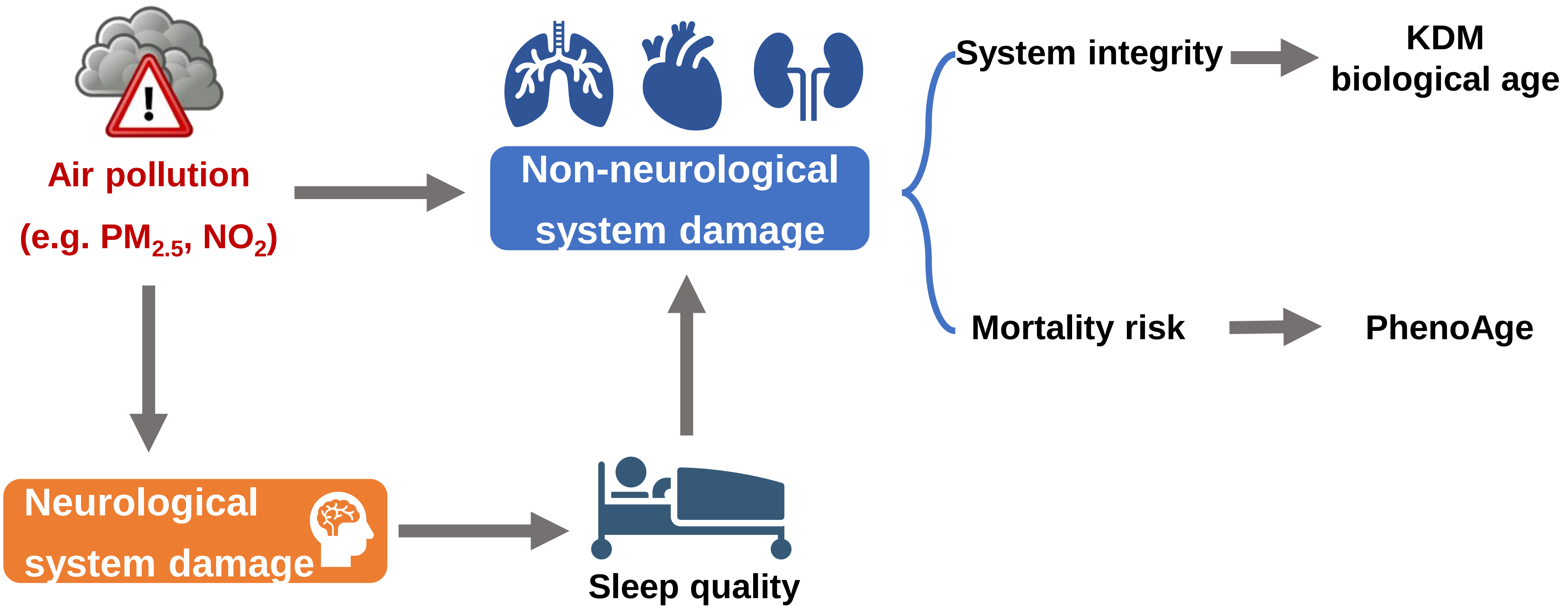

Figure S2 Schematic diagram of the associations between air pollutants, sleep quality, and biological ages
