# Supplementary material for: Role of sleep quality in the acceleration of biological aging and its potential for preventive interaction on air pollution insults: Findings from the UK Biobank cohort
Source: Aging Cell. 2022 Apr 14;21(5):e13610. doi: 10.1111/acel.13610 (PMC9124313; doi:10.1111/acel.13610)
Supplement: Supplementary file 3 — Table S1‐S11 [file ACEL-21-e13610-s002.docx]

**Table S1** Distributions and correlation matrix of the five air pollutants in UK biobank (Pearson correlation)

| **Air pollutants** | **Mean (SD)** | **IQR** | PM_2.5_ | PM_coarse_ | PM_10_ | NO_2_ | NOx |
| --- | --- | --- | --- | --- | --- | --- | --- |
| PM_2.5_ (μg/m³) | 9.96 (1.05) | 1.27 | 1 |  |  |  |  |
| PM_coarse_ (μg/m³) | 6.42 (0.90) | 0.79 | 0.213 | 1 |  |  |  |
| PM_10_ (μg/m³) | 19.23 (2.01) | 2.33 | 0.621 | 0.512 | 1 |  |  |
| NO_2_ (μg/m³) | 28.93 (9.10) | 10.80 | 0.735 | 0.172 | 0.776 | 1 |  |
| NOx (μg/m³) | 43.58 (15.47) | 16.44 | 0.848 | 0.231 | 0.623 | 0.746 | 1 |

All coefficients have a p-value <0.0001; IQR = interquartile range

**Table S2** Associations of six sleep behaviors and sleep index with the accelerations of biological ages in participants that were free of dementia, depression, and anxiety

| **Sleep behaviors** | | KDM-biological age acceleration (years) | |  | PhenoAge acceleration (years) | |
| --- | --- | --- | --- | --- | --- | --- |
|  |  | Coefficients (SE) | *p*-value |  | Coefficients (SE) | *p*-value |
| Self-reported snoring | No | -0.323 (0.023) | **<0.0001** |  | 0.050 (0.049) | 0.23 |
|  | Yes | Ref |  |  | Ref |  |
| Chronotype | Early | -0.085 (0.025) | **0.0007** |  | -0.208 (0.018) | **<0.0001** |
|  | Later | Ref |  |  | Ref |  |
| Frequent daytime sleepiness | No | -0.057 (0.030) | 0.05 |  | -0.118 (0.021) | **<0.0001** |
|  | Yes | Ref |  |  | Ref |  |
| Sleep duration | Normal (7-8h) | -0.205 (0.026) | **<0.0001** |  | -0.124 (0.019) | **<0.0001** |
|  | Short or long | Ref |  |  | Ref |  |
| Insomnia | Never or rarely | -0.028 (0.028) | 0.31 |  | -0.025 (0.020) | 0.20 |
|  | Sometimes or often | Ref |  |  | Ref |  |
| Getting up in morning | Very or fairly easy | -0.015 (0.035) | 0.66 |  | -0.246 (0.025) | **<0.0001** |
|  | Not or not very easy | Ref |  |  | Ref |  |
|  |  |  |  |  |  |  |
| Sleep index (continuous, 0-6) |  | -0.093 (0.010) | **<0.0001** |  | -0.079 (0.007) | **<0.0001** |
| Sleep index (category) | High (5-6) | -0.292 (0.041) | **<0.0001** |  | -0.310 (0.030) | **<0.0001** |
|  | Medium (3-4) | -0.152 (0.038) | **<0.0001** |  | -0.219 (0.027) | **<0.0001** |
|  | Low (0-2) | Ref |  |  | Ref |  |

a: Model adjusted for age, sex, race, BMI, smoking status, healthy alcohol intake, healthy physical activity, years of education (<10 years or ≥10 years), hypertension, diabetes, and coronary heart disease. The examination center was controlled for as a random effect to account for the potential residual bias from examinations. Bolded values that were below the significance threshold, which was 0.05/(8*2) = 0.0031, were considered as statistically significant; Dementia, depression, and anxiety were ascertained using hospital inpatient records and Patient Health Questionnaire (PHQ)-4 questionnaire;

**Table S3** Associations of sleep patterns and biological age accelerations with corresponding genetic risk scores ^a^

| **Trait** |  | **Coefficients** | **SE** | **R-squares (variance explained)** | ***p*-value** |
| --- | --- | --- | --- | --- | --- |
| Sleep patterns | No self-reported snoring (behavior 1) | 0.00351 | 0.000991 | 0.0811 | **0.0004** |
|  | Early chronotype (behavior 2) | 0.00187 | 0.000408 | 0.0257 | **<0.0001** |
|  | No frequent daytime sleepiness (behavior 3) | 0.1318 | 0.0406 | 0.0454 | **0.0012** |
|  | Sleep 7–8 h/day (behavior 4) | 0.00267 | 0.000829 | 0.0225 | **0.0013** |
|  | Never/rarely insomnia (behavior 5) | 0.00559 | 0.000107 | 0.0356 | **<0.0001** |
|  | Getting up easy in morning (behavior 6) | 0.00242 | 0.000965 | 0.0668 | **0.0012** |
|  | Sleep index | 0.01097 | 0.00079477 | 0.0584 | **<0.0001** |
| Biological ages | Acceleration of KDM-biological age | 0.08394 | 0.00471 | 0.5127 | **<0.0001** |
|  | Acceleration of PhenoAge | 0.10881 | 0.00229 | 0.1646 | **<0.0001** |

a: Associations of six sleep patterns with corresponding genetic risk scores were tested by logistic regression models; associations of sleep index and accelerations of both biological ages with corresponding genetic risk scores were tested by linear regression models. Models were adjusted for sex and age. Bolded values that were below the significance threshold, which was 0.05/9 = 0.0056, were considered as statistically significant;

**Table S4** Causal associations between six sleep behaviors and biological age accelerations with unweighted genetic risk scores

| **Sleep patterns** | **Scenarios** | **Estimates** | | | |
| --- | --- | --- | --- | --- | --- |
| No self-reported snoring (behavior 1) | 1. **Sleep pattern → Biological ages** | **KDM-biological age acceleration (years)** | | **PhenoAge acceleration (years)** | |
|  |  | Coefficients (SE) | *p*-value | Coefficients (SE) | *p*-value |
|  | **Observed effects** | -0.286 (0.023) | **<0.0001** | 0.069 (0.067) | 0.26 |
|  | **Genetic-predicted effects** | -5.110 (0.096) | **<0.0001** | 1.107 (0.971) | 0.17 |
|  | **(2) Biological ages → sleep pattern** | **KDM- biological age acceleration (years)** | | **PhenoAge acceleration (years)** | |
|  |  | Coefficients (SE) | *p*-value | Coefficients (SE) | *p*-value |
|  | **Observed effects** | -0.052 (0.004) | **<0.0001** | 0.002 (0.002) | 0.10 |
|  | **Genetic-predicted effects** | 0.030 (0.026) | 0.25 | -0.006 (0.004) | 0.20 |
| Early chronotype (behavior 2) | 1. **Sleep pattern → Biological ages** | **KDM-biological age acceleration (years)** | | **PhenoAge acceleration (years)** | |
|  |  | Coefficients (SE) | *p*-value | Coefficients (SE) | *p*-value |
|  | **Observed effects** | -0.071 (0.022) | **0.0015** | -0.234 (0.017) | **<0.0001** |
|  | **Genetic-predicted effects** | -0.209 (0.164) | 0.20 | -0.218 (0.121) | 0.07 |
|  | **(2) Biological ages → sleep pattern** | **KDM- biological age acceleration (years)** | | **PhenoAge acceleration (years)** | |
|  |  | Coefficients (SE) | *p*-value | Coefficients (SE) | *p*-value |
|  | **Observed effects** | -0.046 (0.005) | 0.0026 | -0.053 (0.004) | **<0.0001** |
|  | **Genetic-predicted effects** | 0.028 (0.027) | 0.30 | -0.004 (0.004) | 0.36 |
| No frequent daytime sleepiness (behavior 3) | 1. **Sleep pattern → Biological ages** | **KDM-biological age acceleration (years)** | | **PhenoAge acceleration (years)** | |
|  |  | Coefficients (SE) | *p*-value | Coefficients (SE) | *p*-value |
|  | **Observed effects** | -0.019 (0.025) | 0.45 | -0.207 (0.019) | **<0.0001** |
|  | **Genetic-predicted effects** | -0.209 (0.047) | **<0.0001** | -0.891 (0.035) | **<0.0001** |
|  | **(2) Biological ages → sleep pattern** | **KDM- biological age acceleration (years)** | | **PhenoAge acceleration (years)** | |
|  |  | Coefficients (SE) | *p*-value | Coefficients (SE) | *p*-value |
|  | **Observed effects** | -0.012 (0.006) | 0.032 | -0.050 (0.005) | **<0.0001** |
|  | **Genetic-predicted effects** | 0.046 (0.024) | 0.05 | 0.004 (0.004) | 0.25 |
| Sleep 7–8 h/day (behavior 4) | 1. **Sleep pattern → Biological ages** | **KDM-biological age acceleration (years)** | | **PhenoAge acceleration (years)** | |
|  |  | Coefficients (SE) | *p*-value | Coefficients (SE) | *p*-value |
|  | **Observed effects** | -0.248 (0.023) | **<0.0001** | -0.220 (0.017) | **<0.0001** |
|  | **Genetic-predicted effects** | -0.323 (0.039) | **<0.0001** | -0.707 (0.029) | **<0.0001** |
|  | **(2) Biological ages → sleep pattern** | **KDM- biological age acceleration (years)** | | **PhenoAge acceleration (years)** | |
|  |  | Coefficients (SE) | *p*-value | Coefficients (SE) | *p*-value |
|  | **Observed effects** | -0.070 (0.005) | **<0.0001** | -0.057 (0.004) | **<0.0001** |
|  | **Genetic-predicted effects** | 0.008 (0.026) | 0.75 | 0.009 (0.004) | 0.046 |
| Never/rarely insomnia (behavior 5) | 1. **Sleep pattern → Biological ages** | **KDM-biological age acceleration (years)** | | **PhenoAge acceleration (years)** | |
|  |  | Coefficients (SE) | *p*-value | Coefficients (SE) | *p*-value |
|  | **Observed effects** | -0.082 (0.023) | **0.0011** | -0.041 (0.019) | 0.028 |
|  | **Genetic-predicted effects** | -1.702 (0.058) | **<0.0001** | -1.341 (0.043) | **<0.0001** |
|  | **(2) Biological ages → sleep pattern** | **KDM- biological age acceleration (years)** | | **PhenoAge acceleration (years)** | |
|  |  | Coefficients (SE) | *p*-value | Coefficients (SE) | *p*-value |
|  | **Observed effects** | -0.019 (0.005) | **0.0004** | -0.009 (0.005) | 0.05 |
|  | **Genetic-predicted effects** | 0.009 (0.024) | 0.70 | 0.005 (0.004) | 0.20 |
| Getting up easy in morning (behavior 6) | 1. **Sleep pattern → Biological ages** | **KDM-biological age acceleration (years)** | | **PhenoAge acceleration (years)** | |
|  |  | Coefficients (SE) | *p*-value | Coefficients (SE) | *p*-value |
|  | **Observed effects** | -0.013 (0.029) | 0.64 | -0.357 (0.021) | **<0.0001** |
|  | **Genetic-predicted effects** | -1.006 (0.044) | **<0.0001** | -1.544 (0.033) | **<0.0001** |
|  | **(2) Biological ages → sleep pattern** | **KDM- biological age acceleration (years)** | | **PhenoAge acceleration (years)** | |
|  |  | Coefficients (SE) | *p*-value | Coefficients (SE) | *p*-value |
|  | **Observed effects** | -0.003 (0.007) | 0.70 | -0.070 (0.005) | **<0.0001** |
|  | **Genetic-predicted effects** | 0.006 (0.021) | 0.77 | -0.001 (0.003) | 0.81 |

a: Effects were estimated by the change in binary sleep behaviors and one SD change in the biological age accelerations. Models were adjusted for age, sex, race, BMI, smoking status, healthy alcohol intake, healthy physical activity, years of education (<10 years or ≥10 years), hypertension, diabetes, and coronary heart disease. The examination center was controlled for as a random effect. The genetic risk scores for each sleep behavior and biological age accelerations were unweighted. Bolded values that were below the significance threshold, which was 0.05/(6*2*2) = 0.0021, were considered as statistically significant;

**Table S5** Causal associations between sleep index and biological age accelerations with weighted genetic risk scores ^a^

| 1. **Sleep index → Biological ages** | **KDM-biological age acceleration (years)** | | **PhenoAge acceleration (years)** | |
| --- | --- | --- | --- | --- |
|  | Coefficients (SE) | *p*-value | Coefficients (SE) | *p*-value |
| **Observed effects** | -0.129 (0.011) | **<0.0001** | -0.153 (0.008) | **<0.0001** |
| **Genetic-predicted effects** | -2.521 (0.048) | **<0.0001** | -0.640 (0.036) | **<0.0001** |
|  |  |  |  |  |
| **(2) Biological ages → sleep index** | **KDM- biological age acceleration (years)** | | **PhenoAge acceleration (years)** | |
|  | Coefficients (SE) | *p*-value | Coefficients (SE) | *p*-value |
| **Observed effects** | -0.034 (0.003) | **<0.0001** | -0.044 (0.002) | **<0.0001** |
| **Genetic-predicted effects** | 0.099 (0.068) | 0.15 | 0.008 (0.011) | 0.48 |

a: Effects were estimated by one SD change in the sleep index or biological age accelerations. Models were adjusted for age, sex, race, BMI, smoking status, healthy alcohol intake, healthy physical activity, years of education (<10 years or ≥10 years), hypertension, diabetes, and coronary heart disease. The examination center was controlled for as a random effect. The genetic risk scores for sleep index and biological ages were weighted by previously reported coefficients. Bolded values that were below the significance threshold, which was 0.05/(2*2*2) = 0.0063, were considered as statistically significant;

**Table S6** Associations of annual average levels of air pollutants with biological ages with and without adjustment for sleep index ^a^

| **Air pollutants** | **Model without sleep index** | |  | **Model with sleep index** | | | |
| --- | --- | --- | --- | --- | --- | --- | --- |
|  |  |  |  | Air pollutants | | Sleep index | |
|  | Coefficients (SE) | *p*-value |  | Coefficients (SE) | *p*-value | Coefficients (SE) | *p*-value |
| KDM-biological age acceleration (years) | | | | | | | |
| PM_2.5_ | 0.060 (0.008) | **<0.0001** |  | 0.056 (0.008) | **<0.0001** | -0.104 (0.009) | **<0.0001** |
| PM_coarse_ | 0.013 (0.015) | 0.37 |  | 0.013 (0.015) | 0.39 | -0.103 (0.009) | **<0.0001** |
| PM_10_ | 0.025 (0.013) | 0.050 |  | 0.020 (0.013) | 0.11 | -0.105 (0.009) | **<0.0001** |
| NO_2_ | 0.097 (0.013) | **<0.0001** |  | 0.094 (0.012) | **<0.0001** | -0.106 (0.009) | **<0.0001** |
| NO_x_ | 0.015 (0.012) | 0.20 |  | 0.012 (0.012) | 0.31 | -0.103 (0.009) | **<0.0001** |
| PhenoAge acceleration (years) | | | | | | | |
| PM_2.5_ | 0.042 (0.006) | **<0.0001** |  | 0.038 (0.006) | **<0.0001** | -0.123 (0.007) | **<0.0001** |
| PM_coarse_ | 0.052 (0.011) | **<0.0001** |  | 0.051 (0.011) | **0.0001** | -0.124 (0.007) | **<0.0001** |
| PM_10_ | 0.098 (0.009) | **<0.0001** |  | 0.093 (0.009) | **<0.0001** | -0.123 (0.007) | **<0.0001** |
| NO_2_ | 0.093 (0.010) | **<0.0001** |  | 0.087 (0.010) | **<0.0001** | -0.122 (0.007) | **<0.0001** |
| NO_x_ | 0.060 (0.008) | **<0.0001** |  | 0.056 (0.008) | **<0.0001** | -0.123 (0.007) | **<0.0001** |

a: Model adjusted for age, sex, race, BMI, smoking status, healthy alcohol intake, healthy physical activity, years of education (<10 years or ≥10 years ), hypertension, diabetes, and coronary heart disease. The examination center was controlled for as a random effect. The coefficients of each air pollutant were demonstrated by one interquartile range increase in air pollutants. Bolded values that were below the significance threshold, which was 0.05/(5*2*2) = 0.0025, were considered as statistically significant;

**Table S7** Joint associations of sleep index and air pollution levels with biological age accelerations ^a^

| **Sleep patterns** | | **Air pollution levels** |  | **KDM-biological age acceleration (years)** | |  | **PhenoAge acceleration (years)** | |
| --- | --- | --- | --- | --- | --- | --- | --- | --- |
|  |  |  |  | Coefficients (SE) | *p*-value |  | Coefficients (SE) | *p*-value |
| **PM_2.5_** | | | | | | | | |
| Sleep index (category) | High (5-6) | Low |  | Ref |  |  | Ref |  |
|  |  | High |  | -0.007 (0.039) | 0.87 |  | -0.035 (0.029) | 0.22 |
|  | Medium (3-4) | Low |  | 0.118 (0.034) | **0.0005** |  | 0.071 (0.025) | **0.0046** |
|  |  | High |  | 0.188 (0.034) | **<0.0001** |  | 0.145 (0.025) | **<0.0001** |
|  | Low (0-2) | Low |  | 0.259 (0.049) | **<0.0001** |  | 0.349 (0.036) | **<0.0001** |
|  |  | High |  | 0.397 (0.047) | **<0.0001** |  | 0.496 (0.035) | **<0.0001** |
| **NO_2_** | | | | | | | | |
| Sleep index (category) | High (5-6) | Low |  | Ref |  |  | Ref |  |
|  |  | High |  | -0.064 (0.040) | 0.11 |  | 0.099 (0.029) | **0.0007** |
|  | Medium (3-4) | Low |  | 0.109 (0.034) | **0.0014** |  | 0.107 (0.025) | **<0.0001** |
|  |  | High |  | 0.192 (0.035) | **<0.0001** |  | 0.250 (0.025) | **<0.0001** |
|  | Low (0-2) | Low |  | 0.286 (0.049) | **<0.0001** |  | 0.413 (0.036) | **<0.0001** |
|  |  | High |  | 0.481 (0.047) | **<0.0001** |  | 0.602 (0.035) | **<0.0001** |

a: Model adjusted for age, sex, race, BMI, smoking status, healthy alcohol intake, healthy physical activity, years of education (<10 years or ≥10 years), hypertension, diabetes, and coronary heart disease. The examination center was controlled for as a random effect. Bolded values that were below the significance threshold, which was 0.05/(2*2) = 0.0125, were considered as statistically significant;

**Table S8** Associations of air pollutants with biological age accelerations by sleep quality ^a^

| **Air pollutants** | **Sleep patterns** | |  | **KDM- biological age acceleration (years)** | | |  | **PhenoAge acceleration (years)** | | |
| --- | --- | --- | --- | --- | --- | --- | --- | --- | --- | --- |
|  |  |  |  | Coefficients (SE) | *p*-value | Interaction *p*-value ^b^ |  | Coefficients (SE) | *p*-value | Interaction *p*-value |
| PM_2.5_ | Sleep index (category) | High (5-6) |  | 0.017 (0.015) | 0.23 | **<0.0001** |  | 0.009 (0.011) | 0.38 | **<0.0001** |
|  |  | Medium (3-4) |  | 0.061 (0.011) | **<0.0001** |  |  | 0.044 (0.008) | **<0.0001** |  |
|  |  | Low (0-2) |  | 0.108 (0.021) | **<0.0001** |  |  | 0.074 (0.016) | **<0.0001** |  |
| NO_2_ | Sleep index (category) | High (5-6) |  | -0.028 (0.023) | 0.10 | **<0.0001** |  | 0.048 (0.017) | **0.0058** | **0.0045** |
|  |  | Medium (3-4) |  | 0.032 (0.018) | 0.051 |  |  | 0.098 (0.013) | **<0.0001** |  |
|  |  | Low (0-2) |  | 0.122 (0.033) | **0.0003** |  |  | 0.133 (0.026) | **<0.0001** |  |

a: Model adjusted for age, sex, race, BMI, smoking status, healthy alcohol intake, healthy physical activity, years of education (<10 years or ≥10 years), hypertension, diabetes, and coronary heart disease. The examination center was controlled for as a random effect. The coefficients of each air pollutant were demonstrated by one interquartile range increase in air pollutants.

b: Interaction p-values were yielded from models with an interaction term, the sleep index category was additionally adjusted in the model along with the interaction term. Bolded values that were below the significance threshold, which was 0.05/(2*2) = 0.0125, were considered as statistically significant;

**Table S9** Associations of air pollutants with biological age accelerations by sleep patterns ^a^

| **Air pollutants** | **Sleep patterns** | |  | **KDM- biological age acceleration (years)** | | |  | **PhenoAge acceleration (years)** | | |
| --- | --- | --- | --- | --- | --- | --- | --- | --- | --- | --- |
|  |  |  |  | Coefficients (SE) | *p*-value | Interaction *p*-value ^b^ |  | Coefficients (SE) | *p*-value | Interaction *p*-value |
| PM_2.5_ | Self-reported snoring | No |  | 0.059 (0.010) | **<0.0001** | **0.0020** |  | 0.035 (0.008) | **<0.0001** | 0.79 |
|  |  | Yes |  | 0.072 (0.015) | **<0.0001** |  |  | 0.049 (0.010) | **<0.0001** |  |
|  | Chronotype | Early |  | 0.056 (0.013) | **<0.0001** | 0.40 |  | 0.031 (0.008) | **<0.0001** | 0.023 |
|  |  | Later |  | 0.061 (0.010) | **<0.0001** |  |  | 0.055 (0.010) | **<0.0001** |  |
|  | Frequent daytime sleepiness | No |  | 0.038 (0.009) | **<0.0001** | **<0.0001** |  | 0.035 (0.007) | **<0.0001** | 0.039 |
|  |  | Yes |  | 0.118 (0.018) | **<0.0001** |  |  | 0.052 (0.013) | **<0.0001** |  |
|  | Sleep duration | Normal (7-8h) |  | 0.031 (0.010) | **0.0017** | **<0.0001** |  | 0.026 (0.007) | **0.0002** | **<0.0001** |
|  |  | Short or long |  | 0.109 (0.015) | **<0.0001** |  |  | 0.067 (0.011) | **<0.0001** |  |
|  | Insomnia | Never or rarely |  | 0.055 (0.009) | **<0.0001** | 0.22 |  | 0.013 (0.012) | 0.28 | **0.0001** |
|  |  | Sometimes or often |  | 0.067 (0.018) | **0.0001** |  |  | 0.050 (0.007) | **<0.0001** |  |
|  | Getting up in morning | Very or fairly easy |  | 0.050 (0.018) | 0.0049 | 0.33 |  | 0.027 (0.015) | 0.07 | 0.95 |
|  |  | Not or not very easy |  | 0.060 (0.009) | **<0.0001** |  |  | 0.043 (0.007) | **<0.0001** |  |
| NO_2_ | Self-reported snoring | No |  | 0.013 (0.016) | 0.41 | 0.17 |  | 0.087 (0.012) | **<0.0001** | 0.57 |
|  |  | Yes |  | 0.034 (0.024) | 0.16 |  |  | 0.099 (0.016) | **<0.0001** |  |
|  | Chronotype | Early |  | -0.005 (0.021) | 0.83 | 0.28 |  | 0.082 (0.012) | **<0.0001** | 0.17 |
|  |  | Later |  | 0.016 (0.017) | 0.35 |  |  | 0.106 (0.016) | **<0.0001** |  |
|  | Frequent daytime sleepiness | No |  | -0.042 (0.015) | 0.0053 | **<0.0001** |  | 0.082 (0.011) | **<0.0001** | 0.24 |
|  |  | Yes |  | 0.159 (0.028) | **<0.0001** |  |  | 0.109 (0.020) | **<0.0001** |  |
|  | Sleep duration | Normal (7-8h) |  | 0.058 (0.016) | **0.0003** | **<0.0001** |  | 0.065 (0.012) | **<0.0001** | **<0.0001** |
|  |  | Short or long |  | 0.152 (0.023) | **<0.0001** |  |  | 0.142 (0.018) | **<0.0001** |  |
|  | Insomnia | Never or rarely |  | 0.005 (0.015) | 0.74 | 0.26 |  | 0.048 (0.019) | **0.0109** | **0.0013** |
|  |  | Sometimes or often |  | 0.032 (0.028) | 0.26 |  |  | 0.107 (0.011) | **<0.0001** |  |
|  | Getting up in morning | Very or fairly easy |  | 0.009 (0.015) | 0.53 | 0.94 |  | 0.069 (0.023) | **0.0033** | 0.45 |
|  |  | Not or not very easy |  | 0.011 (0.029) | 0.71 |  |  | 0.094 (0.011) | **<0.0001** |  |

a: Model adjusted for age, sex, race, BMI, smoking status, healthy alcohol intake, healthy physical activity, years of education (<10 years or ≥10 years), hypertension, diabetes, and coronary heart disease. The examination center was controlled for as a random effect. The coefficients of each air pollutant were demonstrated by one interquartile range increase in air pollutants.

b: Interaction p-values were yielded from models with an interaction term, the category of each sleep behavior was additionally adjusted in the model along with the interaction term. Bolded values that were below the significance threshold, which was 0.05/(2*6) = 0.0042, were considered as statistically significant;

**Table S10** Causal associations between sleep index and biological age accelerations with genetic risk scores retrieved from the GWAS of DNA methylation age ^a^

| 1. **Sleep index → Biological ages** | **KDM-biological age acceleration (years)** | | **PhenoAge acceleration (years)** | |
| --- | --- | --- | --- | --- |
|  | Coefficients (SE) | *p*-value | Coefficients (SE) | *p*-value |
| **Observed effects** | -0.129 (0.011) | <0.0001 | -0.153 (0.008) | <0.0001 |
| **Genetic-predicted effects** | -2.390 (0.047) | <0.0001 | -0.606 (0.035) | <0.0001 |
|  |  |  |  |  |
| **(2) Biological ages → sleep index** | **KDM- biological age acceleration (years)** | | **PhenoAge acceleration (years)** | |
|  | Coefficients (SE) | *p*-value | Coefficients (SE) | *p*-value |
| **Observed effects** | -0.034 (0.003) | <0.0001 | -0.044 (0.002) | <0.0001 |
| **Genetic-predicted effects** | 0.613 (0.237) | 0.0099 | -0.345 (0.544) | 0.53 |

a: Effects were estimated by one SD change in the sleep index or biological ages. Models were adjusted for age, sex, race, BMI, smoking status, healthy alcohol intake, healthy physical activity, years of education (<10 years or ≥10 years ), hypertension, diabetes. The examination center was controlled for as a random effect. The genetic risk scores for sleep index and biological ages were unweighted. The genetic risk score of both biological ages was retrieved from a meta-analysis of GWAS on the DNA methylation age [43].

**Table S11** Full names and field IDs of variables for the construction of sleep index and biological ages

| **Outcomes** | **Labels in the current study** | **Full name in UK Biobank data dictionary** | **Field ID** |
| --- | --- | --- | --- |
| Sleep index | Snoring | Snoring | 1210 |
|  | Chronotype | Morning/evening person (chronotype) | 1180 |
|  | Daytime sleepiness | Daytime dozing / sleeping | 1220 |
|  | Sleep duration | Sleep duration | 1160 |
|  | Insomnia | Sleeplessness / insomnia | 1200 |
|  | Difficulty levels of getting up in the morning | Getting up in morning | 1170 |
| Biological ages | FEV_1_ (L) | Forced expiratory volume in 1-second (FEV_1_) | 3063 |
|  | SBP (mm Hg) | Systolic blood pressure, automated reading | 4080 |
|  | Total Cholesterol (mg/dL) | Cholesterol | 30690 |
|  | Glycated hemoglobin (%) | Glycated haemoglobin (HbA1c) | 30750 |
|  | Blood urea nitrogen (mg/dL) | Urea | 30670 |
|  | Lymphocyte (%) | Lymphocyte percentage | 30180 |
|  | Mean cell volume (fL) | Mean sphered cell volume | 30270 |
|  | Serum glucose (mg/dL) | Glucose | 30740 |
|  | Red cell distribution width (%) | Red blood cell (erythrocyte) distribution width | 30070 |
|  | White blood cell count (1000 cells/uL) | White blood cell (leukocyte) count | 30000 |
|  | Albumin (g/dL) | Albumin | 30600 |
|  | Creatinine (mg/dL) | Creatinine | 30700 |
|  | C-reactive protein (mg/dL) | C-reactive protein | 30710 |
|  | Alkaline phosphatase (U/L) | Alkaline phosphatase | 30610 |
