# Supplementary material for: Role of sleep quality in the acceleration of biological aging and its potential for preventive interaction on air pollution insults: Findings from the UK Biobank cohort
Source: Aging Cell. 2022 Apr 14;21(5):e13610. doi: 10.1111/acel.13610 (PMC9124313; doi:10.1111/acel.13610)
Supplement: Supplementary file 5 — Supplementary Material [file ACEL-21-e13610-s005.docx]

**Supplement methods**

**1. Assessment of sleep behaviors according to UK Biobank questionnaire**

(1) Information on snoring was collected by asking ‘Does your partner or a close relative or friend complain about your snoring?’ with responses of (a) yes or (b) no.

(2) Chronotype was assessed using the following question, ‘Do you consider yourself to be (a) definitely a “morning” person, (b) more a “morning” than “evening” person, (c) more an “evening” than “morning” person, or (d) definitely an “evening” person?’.

(3) Daytime sleepiness was retrieved from the question ‘How likely are you to doze off or fall asleep during the daytime when you don’t mean to? (e.g. when working, reading, or driving)’ with responses of (a) never/rarely, (b) sometimes, (c) often, or (d) all of the time.

(4) Sleep duration was retrieved from the reported hours of sleep by asking ‘About how many hours sleep do you get in every 24 h? (include naps)’.

(5) Insomnia symptoms were obtained by asking ‘Do you have trouble falling asleep at night or do you wake up in the middle of the night?’ with responses of (a) never/rarely, (b) sometimes, or (c) usually.

(6) Difficulty levels of getting up in the morning were assessed by asking ‘On an average day, how easy do you find getting up in the morning?’ with responses of (a) not at all easy, (b) not very easy, (c) fairly easy, or (d) very easy.

**2. Land Use Regression (LUR) model**

LUR models calculate the spatial variation of annual average air pollutant concentration at participants’ home addresses given at the baseline visit, using the predictor variables obtained from the Geographic Information System such as traffic, land use, and topography. Leave-one-out cross-validation showed good model performance for PM_2.5_, PM_10_, NO_2_ and NO_x_ (cross-validation R^2^=77%, 88%, 87% and 88%, respectively) and a comparatively moderate performance for PM_coarse_ (cross-validation R^2^=57%) in the southeast England area (London/Oxford). Details on the development and validation of the ESCAPE LUR models have been described elsewhere (Beelen et al. 2013; Eeftens et al. 2012). The LUR estimates of PM were valid for 400 km from Greater London but not beyond, and as such, patients living in northern England and Scotland were eliminated from the PM analyses.

**References**

Beelen R, Hoek G, Vienneau D, Eeftens M, Dimakopoulou K, Pedeli X, et al. 2013. Development of no2 and nox land use regression models for estimating air pollution exposure in 36 study areas in europe – the escape project. Atmospheric Environment 72:10-23.

Eeftens M, Beelen R, de Hoogh K, Bellander T, Cesaroni G, Cirach M, et al. 2012. Development of land use regression models for pm(2.5), pm(2.5) absorbance, pm(10) and pm(coarse) in 20 european study areas; results of the escape project. Environ Sci Technol 46:11195-11205.
